# Correlation between Periodontitis and Onset of Alzheimer’s Disease: A Literature Review

**DOI:** 10.3390/dj12100331

**Published:** 2024-10-17

**Authors:** Antonio Barbarisi, Valeria Visconti, Dorina Lauritano, Francesca Cremonini, Gianluigi Caccianiga, Saverio Ceraulo

**Affiliations:** 1 Department of Medicine and Surgery, University of Milano-Bicocca, 20100 Monza, Italy; 2 Private Practitioner, 20900 Monza, Italy; 3 Department of Translational Medicine, University of Ferrara, 44121 Ferrara, Italy; 4 Postgraduate School of Orthodontics, University of Ferrara, 44121 Ferrara, Italy; 5 Fondazione IRCCS San Gerardo dei Tintori, 20900 Monza, Italy

**Keywords:** periodontal disease, Alzheimer’s disease, cognitive impairment

## Abstract

Background: Alzheimer’s disease is a slowly progressing neurodegenerative illness and the most common form of dementia. This pathology leads to an increase in cognitive decline and is responsible, in patients, for several difficulties in performing various activities of daily living, such as oral hygiene. Several experimental studies have shown that oral health in patients with Alzheimer’s disease worsens in direct proportion to the progression of the disease due to the appearance of gingivitis and periodontitis. Methods: This clinical literature review aims to evaluate a possible correlation between periodontal disease and Alzheimer’s disease, trying to understand if the periopathogens can contribute to the onset or the progression of Alzheimer’s disease (AD). The study was conducted on the database PubMed (MEDLINE) of full-text systematic reviews in English on humans and animals that were published in the last five years, from 2018 to 2023. This returned 50 publications, which, once the eligibility criteria were applied, resulted in the 10 publications examined in this review. The selected articles were organized through the construction of tables, analyzed, and compared through Judith Garrard’s Matrix method to arrive at the review results. Results: Infection by periopathogens can increase the risk of developing Alzheimer’s disease, but also the onset of the latter can make it more difficult to maintain proper oral hygiene, favoring the onset of periodontal disease: it is possible to affirm the existence of a correlation between periodontitis and AD. It was found that patients exposed to chronic periodontitis have a greater risk of developing a cognitive decline or AD and that oral pathogens can be responsible for neuropathologies and increasing systemic inflammation. Conclusions: Periodontitis and periodontal pathogens represent a real risk factor for the onset or worsening of AD; however, the pathogenetic mechanism is still not completely clear.

## 1. Introduction

Periodontitis is an irreversible inflammatory process that affects the periodontium, i.e., the supporting tissues of the tooth, caused by bacterial pathogens in dental plaque attached to the surfaces of the oral cavity [1]. Its progression leads to bone loss and is the main cause of edentulism among adults.

Periodontitis promotes a chronic and progressive local inflammatory response starting from a reversible inflammation of the more superficial gingival tissue (gingivitis) up to the irreversible destruction of the deep periodontium and the loss of dental elements (PD) [2]. Gingivitis signs are gingival bleeding, redness of the gingiva, gingiva enlargement, and an increase in crevicular fluid, while if they are not treated, gingivitis progresses to periodontitis, with pocket formation, loss of connective tissue attachment, tooth mobility, gingival recession, and bone loss [3,4]. Bacteria colonize teeth forming a structure called biofilm which activates a patient’s immunological response with an increase in inflammatory mediators [5,6]. The balance between microbes and the immune system occurs in all biological systems and this interaction is particularly complex in the oral cavity. In a healthy state, the oral microbiota is composed of commensal microorganisms that live in symbiosis. In a pathological state, however, the host’s microbial networks lead to oral dysbiosis linked to many local and systemic diseases, including periodontitis [7].

Poor oral health, including dental caries, periodontal disease, or complete tooth loss, affects 3.5 billion people [8]. It is an age-related disease: its global prevalence is about 5% to 35 years, 50% to 45 years, and 60% over 45 years [9,10,11,12,13]. A survey of the Italian population conducted between 2016 and 2020 found that, although awareness of periodontitis has increased in recent years, the disparity between periodontal patients and the diagnosis of the disease is still very high. An aggressive form of periodontitis very often requires complex prosthetic treatment with the insertion of dental implants, which involves high costs for patients. Therefore, it is recommended to adopt clinical guidelines focused on evidence-based medicine to improve patient safety in the diagnosis and treatment of periodontitis and to reduce the impact that periodontal disease has on the individual both in social terms of aesthetics and functional discomfort in phonation and chewing, and in economic terms, reducing the expenditure necessary to rehabilitate the oral apparatus following tooth loss [14]. There are many risk factors associated with periodontitis, supported by many studies and they are divided into two main groups: modifiable factors and non-modifiable factors. Non-modifiable factors include genetics, age, gender, and ethnicity while the modifiable ones include local modifiable risk factors such as tooth malposition, which can be treated with an orthodontic treatment, and those lifestyle and health-related risk factors that can be decreased by modifying the outcome, i.e., smoking, diabetes, obesity, stress, nutritional and vitamin deficiencies, alcohol, osteoporosis, medication, cardiovascular disease and the oral microbiota [15].

The etiology of periodontitis highlights the fundamental role played by bacteria in the buccal cavity. The identification of these micro-organisms subsequently allowed their classification into organized complexes using the criteria of Socransky who, following microbiological analyses of the periodontal pockets of subjects suffering from various degrees of periodontitis in a study of 13,261 samples, divided oral bacteria into six complexes by assigning them a color which could be present at the same time [16].

The purple, green, and yellow clusters include the bacterial species that initially colonize the tooth and precede the arrival and multiplication of Gram-negative anaerobic bacteria. These constitute the orange and red clusters and include the species believed to be the most periodontopathogenic [17].

The transition from periodontal health to pathological status is, in most cases, due to an increase in the bacterial population of the red complex (*T. Forsythia*, *P. Gingivalis*, and *T. Denticola*), the most pathogenic group of bacteria, within the oral ecosystem. Orange and yellow complexes have intermediate pathogenic characteristics while the green complex has harmless characteristics.

*Filifactor alocis* is a Gram-positive obligate anaerobic bacterium and it is a newly appreciated member of the periodontal community [18]: its pathogenic characteristic is the ability to survive in the oxidative stress-rich environment of the periodontal pocket and to alter the microbial community dynamics forming biofilms and interacting with lots of oral bacteria.

Recent studies in the literature affirm that not only bacteria are involved in periodontal disease, but also *Herpesviruses* [19]: they stimulate the release of tissue-destructive inflammatory cytokines and proliferation of periopathogens. They also activate osteoclasts and initiation of immunopathogenic or cytotoxic events [20].

AD is a slowly progressing neurodegenerative illness and it is the most common form of dementia.

Alzheimer’s disease was first described in 1906 by neuropathologist Alois Alzheimer observing the case of a 51-year-old woman suffering from an unknown form of dementia [21]. The discovery of this pathology occurred according to the anatomical study of the patient’s brain, not following the theory of psychoanalytic treatment for mental illnesses.

The autopsy showed an atrophic brain without macroscopic foci. The larger cerebral vessels showed arteriosclerotic changes and there were changes in the neurofibrils, forming bundles, leading to the death of the cells [22].

This pathology leads to an increase in cognitive decline, e.g., memory loss, due to the degeneration of the hippocampus. It is responsible for patient’s difficulties in performing many activities of daily living, including oral hygiene [23]. The increasing aging of the population worldwide and the absence of a cure for this disease have led the World Health Organization (WHO) to list it as a global public health priority [24].

Oral health in patients with Alzheimer’s disease worsens in direct proportion to the progression of the disease due to the appearance of gingivitis and periodontitis, mucositis, and peri-implantitis: elderly people with dementia have extensive plaque formation and many oral health problems related to oral soft tissues, such as gingival bleeding, periodontal pockets, stomatitis, mucosal lesions, and decreased salivation [25]. There is also evidence that poor oral hygiene has a negative impact, not only locally, but also systemically on the patient [26]; recently, Alzheimer’s disease has also been indicated as a possible outcome of chronic exposure to periodontitis as a risk factor [27].

In 2024, the National Institute on Aging and the Alzheimer’s Association (NIAA) [28] presented objective criteria for diagnosis (which should be carried out in symptomatic stages) and staging of AD, including recent advances in biomarkers, to improve clinical care. Syndrome (clinically identified impairment) has been separated from biology (etiology).

Alzheimer’s disease (AD) is defined by its biology with the following implications, it is a biological process that starts with the onset of AD’s neuropathologic changes (ADNPC) while people are still without symptoms.

AD is defined by its unique neuropathologic findings; therefore, detection of AD neuropathologic change by biomarkers (used also in preclinical and mild cognitive impairment stages) is equivalent to diagnosing the disease.

Clinical staging of AD [28] is based on 6 stages:

- Stage 0: Asymptomatic, deterministic gene.
- Stage 1: Asymptomatic, biomarker evidence only.
- Stage 2: Transitional decline: mild detectable change, but minimal impact on daily function.
- Stage 3: Cognitive impairment with early functional impact.
- Stage 4: Dementia with mild functional impairment.
- Stage 5: Dementia with moderate functional impairment.
- Stage 6: Dementia with severe functional impairment.

Early changes in Core 1 biomarkers (amyloid positron emission tomography [PET], approved CSF biomarkers, and precision plasma biomarkers [specifically phosphorylated tau 217]) are attributed to specified amyloid β or AD tauopathy pathways. However, these more generally reflect the presence of ADNPC (i.e., neuritic plaques and fascicles). Abnormal Core 1 biomarker results are sufficient to establish a diagnosis of AD and support clinical decision-making throughout disease progression. Subsequent changes in Core 2 biomarkers (biological fluid and tau PET) can provide prognostic information and, if abnormal, imply that AD is causing the symptoms, which are a result of the disease process and are not necessary to diagnose AD. Abnormality on specific Core 1 biomarkers is sufficient to diagnose AD. The following findings can be used to diagnose AD: amyloid PET; CSF Aβ 42/40, CSF p-tau 181/Aβ 42, CSF t-tau/Aβ 42; or “accurate” plasma assays, where “accurate” can be defined as approved CSF assays in detecting abnormal amyloid PET in the intended-use population. Combinations of Core 1 biomarkers may also be used as diagnostic tests.

Core 2 biomarkers are important but often cannot be used as standalone diagnostic tests for AD. Caution should be used to consider an abnormal Core 2 result as an indicator of the presence of a normal Core 1 result because abnormal amyloid PET (or a biofluid surrogate) is nearly always a prerequisite for neocortical AD tauopathy [29].

The A−T2+ biomarker profile is incompatible with the diagnosis of AD [30]. Two classes of biomarkers are not specific to AD but are important for the progression of AD pathogens. These are N and I biomarkers. MRI can be used to identify N biomarkers that are not AD-specific and may derive from a variety of prior or ongoing brain insults. Cerebrovascular disease (V) is a generic definition that includes different forms of vascular pathology or vascular brain injury, which can be identified by MRI, but these modalities are also not AD-specific [28].

The diagnostic criteria are, in large part, a response to rapid advances in fluid-based biomarkers, especially blood, and the approval of drugs specifically targeting amyloid beta (Aβ) pathology for individuals with early symptomatic AD—specifically mild cognitive impairment and mild dementia [28].

This literature review aims to evaluate a possible correlation between periodontal disease and Alzheimer’s disease, trying to understand if periodontal pathogens can contribute to the onset or the progression of Alzheimer’s disease (AD).

## 2. Materials and Methods

This review followed a precise search strategy.

The first step was to identify a set of key terms to be entered into Pubmed’s (MEDLINE) search bar, the main database for the medical and health sector. This step made it possible to work with precise terms to carry out a targeted survey.

Boolean operators were then used to create a syntax to search for topics as precisely as possible: “Periodontitis AND Alzheimer’s disease”, “Periodontitis AND the pathogenesis of Alzheimer’s disease”, “Oral bacteria AND Alzheimer’s disease”, “Oral subgingival bacteria AND Alzheimer’s disease”, “red complex bacteria AND Alzheimer’s disease”, “red complex bacteria AND pathogenesis of Alzheimer’s disease”, “*Porphyromonas gingivalis* AND Alzheimer’s disease”, “*Porphyromonas gingivalis* AND pathogenesis of Alzheimer’s disease”, “*Fusobacterium nucleatum* AND Alzheimer’s disease”, “*Treponema denticola* AND Alzheimer’s disease” for articles to be examined according to precise inclusion criteria. The first filter consisted of selecting papers published in the last five years, from 2018 to 2023: these are very recent studies with the aim to provide the most recent results of studies on the subject.

Titles and abstracts were analyzed independently by two authors (A.B. and V.V.). If they considered a study inappropriate based on the title and abstract, the study was excluded. A third author (S.C.) resolved any disagreements.

The next step was, in compliance with the previous eligibility criteria of this work, to select full text in English on humans and animals, i.e., systematic and bibliographic reviews discarding all articles that did not coincide with the objective of this work. Only those reviews selected that passed the previous selection criteria were further screened by the same two reviewers (A.B. and V.V.) and any discrepancies at this stage were discussed with the third author (S.C.) for a final decision. Papers were considered admissible only if they met the following inclusion criteria: definition of a specific systematic search strategy; use of internationally recognized databases for their references; analysis of data obtained from descriptive studies, randomized clinical trials, and observational studies (such as cross-sectional, case-control, cohort studies) cited and expressly indicated in the document.

The exclusion criteria were in silica studies, not on human or animal studies, inaccessible title or abstract, not in English, and not enough information about the objective of the review.

Once the research was completed, the results obtained were analyzed and discussed in the following chapters.

This modality was adopted to assess and understand whether direct exposure by periodontal bacterial pathogens or indirect exposure induced by periodontitis causes the onset or progression of cognitive impairment and Alzheimer’s disease: the relationship between periodontitis and oral bacteria of the red complex with AD is an issue for which the literature has provided admissible reviews.

The subsequently selected articles were organized through the construction of tables, analyzed, and compared through Judith Garrard’s Matrix method to arrive at the review results. At last, 10 articles were compared using the Matrix method; the other publications were used for the background. The Matrix method is a fundamental method for conducting a literature review, it consists of reading, analyzing, and writing a summary of scientific articles on a specific argument and then comparing them. The focus is on the hypotheses, scientific methods, results, interpretations, and conclusions of the authors.

## 3. Results

**Figure 1:**
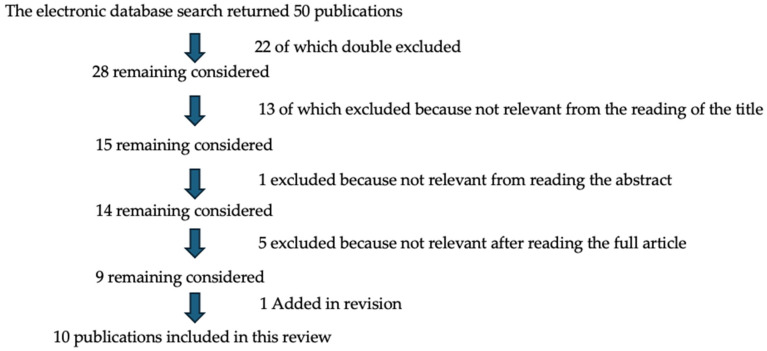
A flowchart of the research process.

The research (Figure 1) has been summarized below.

Based on the research hypothesis, it emerges that:

- review 1: in favor of the research hypothesis.
- review 2: in favor of the research hypothesis.
- review 3: in favor of the research hypothesis.
- review 4: in favor of the research hypothesis.
- review 5: in favor of the research hypothesis.
- review 6: partly in favor of the research hypothesis.
- review 7: in favor of the research hypothesis.
- review 8: in favor of the research hypothesis.
- review 9: in favor of the research hypothesis.
- review 10: in favor of the research hypothesis.

All studies selected (Table 1) are summarized below.

## 4. Discussion

### 4.1. Correlation Between Periodontitis and Alzheimer’s Disease

In the article n°1 [31], a large-scale database search showed that the presence of periodontitis is linked to cognitive decline, suggesting that periodontal pathogens may migrate from their “infectious nidus” to the brain causing inflammation and destruction of brain tissue. Review n°5 [35] found a significant relationship between periodontitis and AD with an odds ratio (OD) of 1.67, which becomes 2.98 in severe forms and a risk ratio (RR) of 1.22; while in article n°7 [37] and n°8 [38] it was found that, after ten years of exposure to periodontitis, the risk of developing AD almost becomes double with an OD of 1.71 compared to healthy people, although it is emphasized that the pathogenetic relationship is still unclear.

Review n°6 [36] also noted a double increase in the risk of developing AD in patients with periodontitis and review n°7 shows an OD of 3.04 in AD patients with severe periodontitis.

In contrast to previous reviews, article n°1 found this correlation after only eight years of exposure to chronic periodontitis and gingivitis and a 2.1-fold increased likelihood of cognitive impairment in patients in whom periodontitis-related bone loss was established by OPT. Review n°7 shows that patients with the most severe forms of periodontitis have lower scores on diagnostic tests for AD.

The latest evidence [41] reinforces the association between PD and AD, describing two pathways for this relationship: microbial involvement and inflammatory cascade. Systemic inflammation in patients with PD can lead to neuroinflammation and periopathogens may contribute to AD progression: patients with an early diagnosis of dementia have a higher risk of developing PD in future [42]. It suggested a bidirectional relationship between the two diseases.

### 4.2. Correlation Between Periodontal Bacteria and Alzheimer’s Disease

In review n°2 [32] and n°9 [39], the presence of *P. Gingivalis* or its gingipains was detected in the brains of mice with periodontitis that caused neuroinflammation and neuronal dysfunction. It has also been shown that more virulent strains of *P. Gingivalis* cause more severe periodontitis, with greater neuroinflammation associated with systemic markers and greater cognitive impairment than less virulent strains, but regardless of their virulence, the strain has been detected in the brain.

In review n°3 [33] it emerged that the presence of *P. Gingivalis* and *T. Denticola* in the oral microbiota correlated with AD while T. Forsythia did not.

In review n°4 [34] it emerged that *P. Gingivalis* and *T. Denticola* exploit periodontal pockets to enter the bloodstream and reach the brain.

As shown in review n°6 [36] the presence of *P. Gingivalis* and gingipains causes neuronal impairment and inflammatory responses typical of AD, although gingipains were not detected in all AD brains.

Review n°7 [37] showed that the presence of *P. Gingivalis*, *Fusobacterium Nucleatum*, *Campylobacter Rectus*, and *Streptococcus Intermedius* correlates with increased mortality from AD over the age of 65, and the co-presence of *P. Gingivalis* and *Campylobacter Rectus* aggravates the risk further.

Review n°8 [38] highlighted that the levels of *Prevotella Intermedius*, *T. Denticola*, and *Fusobacterium Nucleatum* in patients without AD are further increased along with *P. Gingivalis* levels after the onset of AD. Review n°10 [40] noted that the presence of periodontal pathogens and brain abscesses confirms the ability to invade the brain.

Studies have shown that patients with AD have higher microbiome diversity [33,37], with some bacteria that are more present in periodontal patients rather than healthy people (Table 2).

However, some studies [33] highlight that patients with AD differ from patients with other forms of dementia (DEM-noAD); the result is that periodontal pathogens do not act as a trigger for developing AD.

*P. Gingivalis* and *C. Rectus* were linked to an increased risk for incidence of AD over 65 years of age [37].

The presence of *P. Gingivalis*, *P. Intermedia*, *P. Nigrescens*, *F. Nucleatum*, *C. Rectus*, *S. intermedius*, *C. Ochracea*, and *P. Melaninogenica* can be related to increased AD mortality risk above 65 years of age [37].

*S. Oralis* increases the risk of major NCD (all causes) in men, while *E. Corrodens* increases the risk of major NCD (all causes) in women.

This provides evidence that some periodontal pathogens can lead to AD if present, which is even stronger for older adults.

### 4.3. Correlation with the Detection of Antibodies to Periodontal Bacteria and Alzheimer’s Disease

Review n°1 [31] correlated mild cognitive decline with the presence of specific IgG against *P. intermedia*, *F. nucleatum*, *P. gingivalis*, and *T. denticola*.

Review n°2 [32] examined the presence of *P. Gingivalis*, *T. Denticola*, and *T. Forsythia* in the brain and CSF both in patients with and without AD; in addition to their specific antibodies, a sixfold greater impairment was found in patients with AD where *P. Gingivalis* was also present than in patients without. Reviews n°8 [38] and n°10 [40] also found *P. Gingivalis* in the CSF of mice and humans, arguing that this is a clear etiopathogenetic indication.

In review n°3 [33], the presence of specific IgG antibodies against *P. Gingivalis* and the other bacteria of the red–orange cluster was associated with a higher risk of death from AD; patients with higher antibody levels against *Actinomyces Naeslundii* had a higher risk of developing AD, while those with higher antibody levels to *Eubacterium Nodatum* have a lower risk.

Also in review n°3, the presence of *P. Gingivalis* and gingipains in brain tissue and antibodies against *P. Gingivalis*, *T.Forsythia,* and *T. Denticola* were detected in AD patients but not in healthy patients.

In review n°4 [34] it was found that the presence of *Fusobacterium Nucleatum* and *Prevotella Intermedia* is also associated with AD. In the article n°6 [36] significantly elevated levels of IgG against *P. Gingivalis* were detected in 100% of patients with periodontitis and 80% of healthy controls showing that this antibody presence could also be indicative of other factors.

Reviews n°3 and n°7 [37], however, did not reveal a significant relationship between serum antibody levels against *P. Gingivalis* and the rate of cognitive decline, whereas in review n°10 it did.

### 4.4. References to the Blood–Brain Barrier (BBB)

As reported in review n°2 [32], *A. Actinomycetemcomitans* injected into the hearts of mice managed to cross the blood–brain barrier.

Also reviews n°4 [34], n°5 [35], n°6 [36], n°7 [37], n°8 [38], and n°10 [40] showed that *P. Gingivalis* or its LPS and inflammatory mediators and periodontal mediators are able to enter the bloodstream and alter the permeability of the BBB, inducing the deposition of Aβ in response, one of the typical lesions of AD. Alteration of the BBB has been shown to always coincide with AD-related cognitive decline; periodontal pathogens and their factors (but also HSV-1 acting synergistically with *P. Gingivalis*) stimulate a neuroinflammatory response, leading to neurodegeneration [43]. In addition, in review n°7 it was found that the ability of gingipains to break down anti-inflammatory cytokines and go through the neural system further contributes to AD.

**Figure 2:**
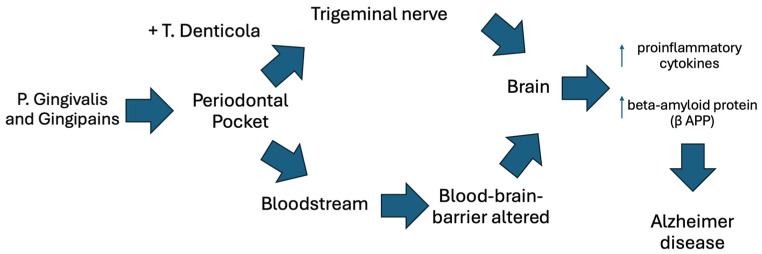
How pathogens spread from the oral cavity to the brain.

Review n°4 [22] showed that the synergism between *P. Gingivalis* and *T. Denticola* also allows them to bypass the BBB colonizing the trigeminal nerve, which provides an alternative route to colonization of the brain (Figure 2).

Some studies [34] demonstrated that *T. Denticola* releases Aβ1–40 and Aβ1–42 from its parent protein (amyloid precursor protein) due to the activation of β-secretase and γ secretase, resulting in the GSK-3β activation and tau phosphorylation; this protein is important for the stabilization of the cytoskeleton of neurons, but in this form, it can contribute to AD.

### 4.5. Correlation Between Periodontitis-Induced Pro-Inflammatory Cytokines and Alzheimer’s Disease

Reviews n°1 [31], n°2 [32], n°3 [33], n°5 [35], n°7 [37], n°8 [38], n°9 [39], and n°10 [40] found the presence of increased levels of pro-inflammatory cytokines IL-1β, IL-6, IL-8, IL-10, PCR, and TNF-α in the brain tissue astrocytes and neurons of human and mouse AD patients in response to the presence of periodontal pathogens including *P. Gingivalis* and LPS from *P. Gingivalis* and gingipains, resulting in neurodegeneration.

Review n°1 also found a reduction in EGF in the same conditions. Review n°9 also claimed that the presence of Aβ, IL-1β, and TNF-α in the brain can be used as markers to indicate inflammation in the CNS.

Review n°10 found an association between increased IL-1β, IL-6, and TNF-α and higher incidence of loss of intellectual capacity in elderly patients.

Review n°1 also found a reduction in EGF. In Reviews n°2, n°9, and n°10, administration of gingipain inhibitors in the brains of mice blocked gingipain-induced neurodegeneration, decreased *P. Gingivalis* levels, and the inflammatory response in the brain by blocking Aβ production.

In review n°2, an increase in TNF-α in the brain was also recorded following injection of *A. Actinomycetemcomitans* into the heart of mice.

Review n°1 found that immune suppression combined with cytokine modulation helps *P. Gingivalis* evade host defenses by exacerbating the AD state.

In review n°3, despite an increased presence of systemic inflammatory markers, no significant relationship with cognitive decline was found.

### 4.6. Limitations

The research gap and future research ideas are shown in the table below (Table 3).

## 5. Conclusions

The aim of this review is to focus attention on the consequences that an untreated form of periodontitis can cause, such as the onset of AD-related cognitive impairment in healthy patients and aggravation in already affected patients.

Over the last five years, this topic has become much debated worldwide, giving rise to several studies; however, it must be pointed out that the different diagnostic and analytical methods have currently placed a limit on the analysis of the results obtained, in many cases not allowing comparison with other homologous studies.

In agreement with the scientific literature and with the results of this review, it is possible to affirm the existence of a correlation between periodontitis and AD; periodontitis and periodontal pathogens constitute a real risk factor for the onset or worsening of AD. These two diseases, which are becoming increasingly common due to the aging of the population, place a burden on healthcare expenditure; it is, therefore, necessary to increase public awareness through primary prevention campaigns and enhance the figure of the dental hygienist as it is their specific task to educate patients on the importance of maintaining proper professional and home oral hygiene.

On the other hand, professionals in the field of odontology should be aware of this social role, especially the dental hygienist who, first and foremost, has the aim of promoting the prevention and treatment of periodontal disease.

In the presence of neglected or long-standing lesions in the oral cavity, specialists responsible for prevention and treatment should alert patients to the potential risk of going on to very disabling systemic diseases, such as AD. During a professional session, the dental hygienist might detect signs of periodontitis and, following a medical diagnosis by a dentist, try to contain it and limit its progression, proposing to the patient or caregiver possible therapeutic and maintenance alternatives, providing the necessary assistance by means of scheduled in-office check-ups during which salivary sampling, laser decontamination, root planing, and indicating the measures that can be applied at home.

It is important to improve the oral hygiene and oral health of older people with AD, and also the oral hygiene education of assistants assigned to the care of elderly patients.

In this regard, the following may be suggested to the patient, or to the caregiver taking care of him/her: to use an electric toothbrush where a manual one has been in use, in order to facilitate plaque removal operations and make them less operator-dependent; a correct brushing technique; the use of toothbrushes; and, finally, that of toothpastes that discourage oral dysbiosis in favor of a balance of the resident microbiota.

As a future perspective, after agreeing on international guidelines on diagnosis and analysis in order to ensure a comparison, it would be desirable to carry out further experimental studies with the aim of establishing not only the bacterial species and inflammatory molecules involved in this process but also their quantities necessary for the initiation of cognitive impairment and the impact of the therapeutic countermeasures taken by professionals in our field to slow or stop the progression of the pathology.

## Figures and Tables

**Table 1:** Articles selected for this work.

N° Article	Authors, Title, Journal, Year of Publication	Objective	Studies and Databases	Conclusions
1	Najwane Said Sadier, Batoul Sayegh, Raymond Farah, Linda Abou Abbas, Rania Dweik, Norina Tang, David M Ojcius, Association between periodontal disease and cognitive impairment in adults [31].	Systematic review with the purpose of evaluating whether there is a greater risk of cognitive deterioration among adult patients with periodontitis with age equal to or greater than 18 years in comparison to adults without periodontitis.	11 observational studies published by September 2021 on PubMed, Web of Science, and CINAHL.	Patients exposed to chronic periodontitis for at least eight years show an increase risk of developing cognitive decline or AD; alveolar bone loss increases 2.1 times the risk of developing cognitive impairment. However, some responsible mechanisms are still unclear and deserve further investigation.
2	Valeria Parra-Torres, Samanta Melgar-Rodríguez, Constanza Muñoz-Manríquez, Benjamín Sanhueza, Emilio ACafferata, Andrea C Paula-Lima, Jaime Díaz-Zúñiga, Periodontal bacteria in the brain-Implication for Alzheimer’s disease: A systematic review [32].	Systematic review with the aim of evaluating the presence of periodontal bacteria in the brain and their role in the debut and progression of AD in humans and animals.	23 observational studies and experiments published between 2002 and 2020 on Medline, Latindex, SciELO, and Cochrane Library databases.	Infection by oral pathogens in animals was related to the development of neuropathological characteristics of AD and the detection of bacteria in the brain. In patients with AD, oral bacteria were detected in brain tissues at pro-cytokines inflammatory increasing levels. Bacteria in the brain are related to pathological characteristics of AD. However, it was discovered that oral bacteria can be detected in the brain even in the absence of periodontitis, therefore the bacterial virulence and the host susceptibility will influence the potential neuroinflammatory response.
3	Samantha Mao, Chen-Pang Huang, Hsin Lan, Hing-Ger Lau, Chun-Pin Chiang, Yi-Wen Chen, Association of periodontitis and oral microbiomes with Alzheimer’s disease: A narrative systematic review [33].	Systematic review with the aim of evaluating the correlation between oral microbiome and development of AD.	26 observational studies published before November 2021 on Pubmed, Embase, and Google Scholar.	Periodontal pathogens play a role in the pathogenesis of AD; however, it would be appropriate to standardize the operating protocol and the sampling site to obtain clearer results. Further studies are also needed to clarify whether periodontal therapies have positive feedback on the prevention and cure of AD.
4	Flavio Pisani, Valerio Pisani, Francesca Arcangeli, Alice Harding, Simarjit Kaur Singhrao, The mechanism pathways of periodontal pathogenesis entering the brain: the potential role of *Treponema denticola* in tracing Alzheimer’s disease pathology [34].	Systematic review with the aim of evaluating the methods of entry of periodontal pathogens to the brain with a focus on *T. Denticola*.	99 observational studies published before July 2022 on Pubmed and Google Scholar.	The spread of *T. Denticola* in the blood circulatory system causes neuro-inflammation of the Trigeminal nerve and, where present, increases the blood–brain barrier permeability favoring the entry of other pathogens into the brain, including *P. Gingivalis*, implicated directly into the lesion formation typical of AD. Furthermore, it highlights the importance of oral hygiene for containment of pathogenic bacteria present.
5	Sriram Kaliamoorthy, Mahendirakumar Nagarajan, Vijayparthiban Sethuraman, Kavitha Jayavel, Vijayalakshmi Lakshmanan, Santosh Palla, Association of Alzheimer’s disease and periodontitis-a systematic review and meta-analysis of evidence from observational studies [35].	Systematic review and meta-analysis with the aim of investigating if adult individuals exposed to periodontitis or periodontal pathogens have a greater probability of developing AD.	6 observational studies published between January 2010 and March 2020 on PubMed, Google Scholar, Web of Science, EMBASE, and Scopus.	Patients with periodontitis present a greater probability of developing AD compared to patients without periodontitis. It highlights the importance of oral hygiene in patients with AD.
6	Abdelrahman Elwishahy, Khatia Antia, Sneha Bhusari, Nkorika Chiamaka Ilechukwu, Olaf Horstick, Volker Winkler, Porphyromonas Gingivalis as a Risk Factor to Alzheimer’s Disease: A Systematic Review [36].	Systematic review with the aim of identifying if exposure to *P. Gingivalis* and its virulence factors increase the risk of the onset of AD.	6 observational studies published before the 31st August 2020 on Cochrane Library, Google Scholar, Ovid, PubMed, Web of Science, and WHOLIS.	The results have not shown a clear association between *P. Gingivalis* and AD; however, the majority of the studies taken into consideration suggest that the bacterium *P. Gingivalis*, through its virulence factors, is important in the process of systematic inflammation. Furthermore, this study revealed heterogeneity in the methodologies of measurement.
7	Leslie Borsa, Margaux Dubois, Guillaume Sacco, Laurence Lupi, Analysis the link between periodontal disease and Alzheimer’s disease: a systematic review [37].	Systematic review and meta-analysis with the primary objective to evaluate the correlation between periodontitis and AD in patients of equal age or over 65 years old and the secondary objective to determine the presence of specific bacterial pathogens involved in periodontal disease.	5 observational studies published between 2010 and June 21, 2021 on PubMed, Cochrane Library, and EMBASE.	The selected studies have found a relationship between periodontitis and AD, denoting that exposure to chronic periodontitis for at least 10 years increases the risk of occurrence of AD by 1.7 times; in patients with AD it was found that periodontopathogenic bacteria associated with a higher risk, high incidence, or mortality due to AD including *P. Gingivalis*, *Campylobacter Rectus*, and *Fusobactrium Nucleatum*. Further studies are necessary to better understand the etiology and the mechanisms of this interaction. A better understanding will permit the improvement of effective prevention measures and even care. The treatment of pockets, therefore, could represent a strategy for managing AD.
8	C. Lorenzi, N. Bianchi, A. Pinto, V. Mazzetti, C. Arcuri, Association of Alzheimer’s The role of periodontal bacteria, Porphyromonas gingivalis, in Alzheimer’s disease pathogenesis and aggravation: a review [38].	Systematic review with the purpose of establishing the role of periodontal bacteria, especially in *P. Gingivalis*, in the pathogenesis of AD.	9 observational studies published between 2016 and May 2021 on PubMed, Cochrane Library, and Web of Science.	The correlation between periodontal bacteria and AD is confirmed; however, the pathogenetic mechanisms are still not completely clear. In the studies, the presence of *P. Gingivalis* was detected in the brain and the CSF of AD patients; the patients affected by chronic periodontitis present an increase in concentration of IL-6 e TNF-α. A study states that ten-year exposure to periodontitis increases the risk of developing AD by 1.7 times.
9	Moan Jéfter Fernandes Costa, Isabela Dantas Torres de Araújo, Luana da Rocha Alves, Romerito Lins da Silva, Patricia Dos Santos Calderon, Boniek Castillo Dutra Borges, Ana Rafaela Luz de Aquino Martins, Bruno Cesar de Vasconcelos Gurgel, Ruthineia Diogenes Alves Uchoa Lins, Relationship of *Porphyromonas gingivalis* and Alzheimer’s disease: a systematic review of pre-clinical studies [39].	Systematic review with the purpose of evaluating whether the animals infected with *P. Gingivalis* are more affected by AD compared to healthy ones.	9 observational studies and published experiments between 2015 and 2019 on PubMed, LILACS, SciELO, ScienceDirect, Web of Science, Cochrane, and SCOPUS.	In animals, a correlation is amply demonstrated between AD and infection with Pg-LPS or *P. Gingivalis*, also mediated from the gingipains, which activates the waterfall complement, increases the production of Aβ and enhances the expression of pro-inflammatory cytokines, causing cerebral inflammation, neuroinflammation, and neurodegeneration, promoting cognitive deterioration. The analyzed studies indicate that periodontitis also has a harmful effect on the development and progression of AD.
10	Mario Dioguardi, Vito Crincoli, Luigi Laino, Mario Alovisi, Diego Sovereto, Filiberto Mastrangelo, Lucio Lo Russo, Lorenzo Lo Muzio, The role of periodontitis and periodontal bacteria in the onset and progression of Alzheimer’s Disease [40].	Systematic review with the purpose of evaluating the correlation between periodontal bacteria and AD.	15 observational studies published between 1989 and 2019 on PubMed and SCOPUS.	The analysis of the literature of scientific evidence shows that periodontitis can contribute to inflammation of the peripheral environment through the introduction of periodontal bacteria and pro-inflammatory cytokines favoring the onset of AD; however, further careful investigations on periodontal pathogens are necessary.

**Table 2:** Predominant microbes in AD patients vs. healthy patients.

AD Patients	Healthy Patients
*Slackia exigua*	*Actinomyces*
*Lachnospiraceae*	*Rothia*
*Prevotella oulorum*	
*Moraxella*	
*Leptotrichia*	
*Sphaerochaeta*	
*F. Nucleatum*	

**Table 3:** Limitations of the study.

Research Gap	Future Research Ideas
Absence of univocal results	To establish international guidelines on diagnosis and methods of analysis
Poor statistical analysis to better demonstrate the correlations between AD and PD	Experimental studies with the aim of establishing not only the bacterial species and inflammatory molecules involved in this process but also their quantities necessary for the initiation of cognitive impairment
Data not sufficient to relate this correlation to the age and sex of the patients	Experimental studies with the aim of establishing the impact of the therapeutic countermeasures taken by professionals in our field to slow or stop the progression of the pathology
Different diagnostic methods, analytical methods, and definition of periodontitis did not permit a very detailed comparison of the studies because they were not homologous	Further studies with a unique definition of periodontitis, following the 2017 World Workshop on the Classification of Periodontal and Peri-Implant Diseases and Conditions are needed, to control for confounding variables

## Data Availability

Data from this study are available upon reasonable request by writing to the corresponding.
